# Multisensory Tracking of Objects in Darkness: Capture of Positive Afterimages by the Tactile and Proprioceptive Senses

**DOI:** 10.1371/journal.pone.0150714

**Published:** 2016-03-09

**Authors:** Brian W. Stone, Jessica Tinker

**Affiliations:** Department of Psychology, University of Georgia, Athens, Georgia, United States of America; Centre de Neuroscience Cognitive, FRANCE

## Abstract

This paper reports on three experiments investigating the contribution of different sensory modalities to the tracking of objects moved in total darkness. Participants sitting in the dark were exposed to a brief, bright flash which reliably induced a positive visual afterimage of the scene so illuminated. If the participants subsequently move their hand in the darkness, the visual afterimage of that hand fades or disappears; this is presumably due to conflict between the illusory visual afterimage (of the hand in its original location) and other information (e.g., proprioceptive) from a general mechanism for tracking body parts. This afterimage disappearance effect also occurs for held objects which are moved in the dark, and some have argued that this represents a case of body schema extension, i.e. the rapid incorporation of held external objects into the body schema. We demonstrate that the phenomenon is not limited to held objects and occurs in conditions where incorporation into the body schema is unlikely. Instead, we propose that the disappearance of afterimages of objects moved in darkness comes from a general mechanism for object tracking which integrates input from multiple sensory systems. This mechanism need not be limited to tracking body parts, and thus we need not invoke body schema extension to explain the afterimage disappearance. In this series of experiments, we test whether auditory feedback of object movement can induce afterimage disappearance, demonstrate that the disappearance effect scales with the magnitude of proprioceptive feedback, and show that tactile feedback alone is sufficient for the effect. Together, these data demonstrate that the visual percept of a positive afterimage is constructed not just from visual input of the scene when light reaches the eyes, but in conjunction with input from multiple other senses.

## Introduction

The brain uses information from multiple sensory modalities to track the spatial location of objects, including body parts. When those senses provide conflicting information—as when visual input tells me something is happening *here* and auditory input tells me it is happening *there*—the brain must weigh that incoming information if it is to establish a unified percept of the object's spatial location [1–2]. In practice, one sense will often dominate and is said to 'capture' the other senses; which sense overrules the others depends on the context. In many well-studied circumstances, vision captures other senses, as with prism glasses [3] (see also [4]), the Ventriloquist Illusion [5], and the Rubber Hand Illusion [6]. However, vision can also be captured by another sense, as found in work with positive afterimages.

A bright flash can induce a positive visual afterimage of whatever scene a person is looking at when the flash occurs. Aspects of this afterimage change as a result of the person subsequently moving their body in the dark relative to the afterimaged scene or moving afterimaged body parts relative to the rest of the scene [7–9]. For example, an afterimage of one's hand tends to disappear, fade, or 'crumble' if the hand is subsequently moved in the darkness, suggesting that the appearance of positive afterimages is not solely due to retinal processes, but involves central interaction between vision and other sensory information like proprioception [8, 10] (see also [11–12]). Proprioception signals that the limb has moved, despite the persistence of (illusory) visual information from the afterimage signaling that the limb is in the original location it occupied when the flash occurred. This sensory conflict is resolved by the immediate disappearance of the visual afterimage of the moved body part (but not the rest of the afterimage); that is, proprioception captures vision.

Carlson et al. [13] demonstrated that this same effect occurs when an observer moves a held object in the dark after being exposed to a bright flash. The observer experiences an afterimage of the scene (both hand and held object), and subsequently moving the held object in the dark causes the afterimage of the moved object to disappear or fade. This happens whether the held object is moved along with a moving limb or simply from being dropped in the dark, and even occurs when the participant is not holding the object during induction of the afterimage, but simply reaches out and grabs the object (in the dark) after an afterimage of it has formed. Since the disappearance of afterimages of moved limbs is thought to be due to proprioceptive updating of bodily representations, Carlson et al. [13] interpret these results as evidence that held objects are rapidly assimilated or incorporated into an extended body representation.

When the object was held in a mechanical gripper and subsequently dropped by relaxing the gripper handle, Carlson et al.'s [13] participants did not report that the afterimage of the object disappeared. Clearly knowledge of the object's change of location was not sufficient to induce a change in the afterimage and participants were not simply reporting object disappearance in every condition. Beyond that, the condition also seems to show that in this paradigm, second-order extensions of the body do not happen; the gripper may become part of the body (this was not tested directly), but an object held in another held object does not. More recent work found afterimage fading for an object dropped from a freely-held gripper tool, but not from gripper tool affixed to a table [14]. This fading in a freely-held tool condition has been interpreted as a second-order extension of the body schema [15] and a short-lived change in the brain's representation of the body [16].

Whether first-order or second-order, this rapid extension of the body schema to include held (or indirectly-held) objects is apparently at odds with previous work on the prerequisite conditions and time scale of body schema extension. Ikiri et al. [17] seem to show that use of a rake tool in monkeys extended the visual receptive field of neurons representing peripersonal space (i.e. extended the neural representation of bodily boundaries), yet this required weeks of training (also, see [18] for methodological concerns about this study). Behavioral work with humans requires significant training before the onset of effects that are taken as evidence of peripersonal space extension [19]. The Rubber Hand Illusion—the paradigm with the strongest evidence for incorporation of external objects into the body schema—generally only works with objects which look like real limbs, in an anatomically plausible posture [20–23] (cf [24]). So if clearly non-corporeal objects are being incorporated into the body schema during the brief duration in which the object is held, it would represent a very impressive–perhaps even worrisome–flexibility of the body schema.

Carlson et al. [13] acknowledge that their body schema extension account of the afterimage effect is apparently at odds with this prior literature, but suggest there may be both long-term, durable representations of the body as well as a highly plastic representation that can assimilate new objects within seconds, despite little or no training. Rademaker et al. [16] built on this idea, showing that the afterimage of cotton balls dropping from freely held chopsticks fades in a minority of trials (26%) when dropped in darkness, but furthermore demonstrating that those skilled with chopsticks were more likely to experience the effect when dropping from their dominant hand, and that long-term training with chopsticks increased the effect markedly. However, they admit that the body schema integration interpretation is tentative and that their results are not proof of integration into the body schema [16].

While the disappearance of an afterimage of a moved body part has been framed as a byproduct of a multi-sensory mechanism for tracking limbs (and this interpretation drives the body schema extension account of object fading), we present evidence that this may instead represent a special case of a more general system for tracking objects—including non-bodily objects—by integrating information from multiple senses, and this system functions in darkness as in daylight. When an object is moved or dropped in the dark, there are potential tactile, proprioceptive, auditory and reafferent motor signals which could update the brain about the changed location of the object despite a complete lack of new visual information (indeed, despite the illusory persistence of visual information that the object has not moved). As we shall see, these changes appear to happen automatically as a result of the brain weighing information from multiple senses to track the object in space.

In the three experiments presented herein, we directly test this sensory-updating explanation and provide evidence that the visual afterimage percept disappears as a result of non-visual sensory feedback even in circumstances where body schema incorporation is very unlikely.

In the first experiment, we demonstrate that the disappearance effect happens even when the object is not held, suggesting body schema extension is either extremely liberal or unnecessary to explain the effect. Furthermore, we test whether the effect is influenced by auditory feedback of an object dropped in the darkness (either directly from the hand or released from a gripper tool). Previous studies either did not mention auditory feedback [14] or attempted to minimize or remove it by having participants drop wool [13] or cotton balls [16]. We had participants drop an object with loud auditory feedback (wooden block into metal bowl) or minimal auditory feedback (wooden block into suspended padding in a bowl); we did this both for participants dropping held objects and for participants dropping objects from a freely-held gripper tool. Unfortunately, the results involving auditory manipulations were inconclusive, possibly due to residual auditory information in the quiet conditions and/or due to an interaction between audition and proprioception.

Thus, in a second experiment, we remove any residual auditory information by using noise-cancelling headphones and test whether the disappearance effect is indeed influenced by auditory signals occurring well after any physical contact has ceased. We also test a control condition of no movement.

Finally, in a third experiment, we clearly demonstrate that the disappearance effect scales with the magnitude of proprioceptive feedback by having participants drop objects of differing mass (while controlling for auditory feedback). We also demonstrate that passive tactile feedback is sufficient to induce the effect, further undermining the idea that holding an object or initiating a motor action is necessary. Together, these three experiments elucidate the role that the various senses play in establishing a multi-modal representation of object location in darkness, implicating proprioceptive, tactile, and possibly auditory information as contributors.

## Experiment 1—Method

### Participants

Fourteen healthy adult undergraduate and graduate student volunteers (8 female, 6 male) participated in this experiment. Age ranged from 20 to 34 years (mean = 28.4). All were right handed and all had normal or corrected-to-normal vision. Participants were asked to remove any watches and jewelry from their hands and wrists prior to starting the experiment. All participants were naïve as to the hypotheses of the experiment. All research was approved by the Institutional Review Board of the University of Georgia; each participant signed a consent form and was debriefed after the study.

### Materials and apparatus

During testing, the entire room was completely dark. Participants sat at a table (155 x 79 cm) which, along with the background visual field, was covered by a non-reflective black cloth. A Dynalite Uni400JR flashbulb (400 w-s) was mounted 230 cm behind and 90 cm to the left of the participant and 2 m above the ground. The experimenter remained 2.5 m behind the participant at all times, to discharge the flash and to enter a small 90 x 90 cm light-sealed booth between trials to record responses and give instructions for upcoming trials.

**Fig 1:**
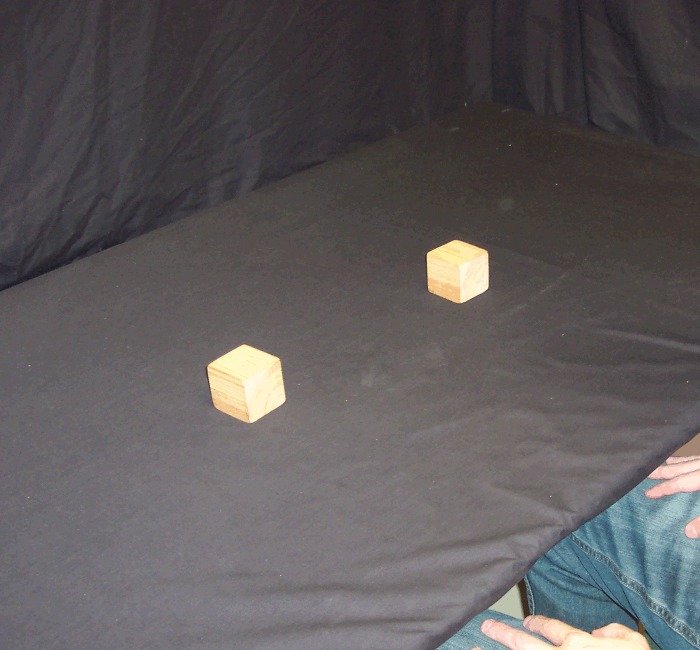
Blocks on the table. Blocks placed on the table 30 cm apart, at arm's reach.

**Fig 2:**
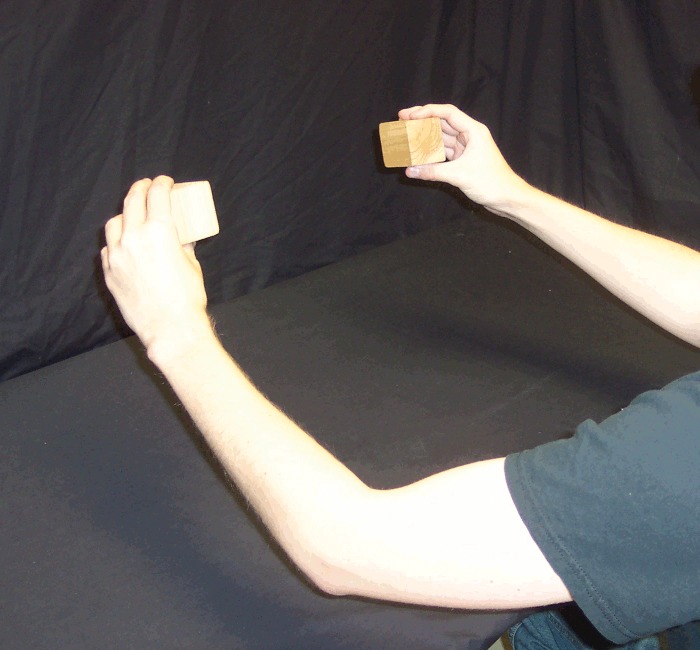
Blocks held by participant. Participant holding blocks approximately 20 cm above the table.

For each trial, a pair of cubic wooden blocks (5 x 5 x 5 cm, weighing 60 g) was either placed on the table approximately 30 cm apart, just within arm's reach equidistant in front of the participant, or held (by the participant) at the same distance but approximately 20 cm above the table (Figs 1 and 2). Although Carlson et al. [13] used wool in order to minimize auditory feedback when dropped, we chose an object which could be dropped in such a way that it either does or does not make noise (i.e. it allows a loud condition as well as a quiet condition).

Throughout each trial, the participant was instructed to fixate directly forward at the midpoint between the starting location of the two blocks. Thus, for trials where the blocks were placed 30 cm apart on the table, the participant fixated at the point on the table mid-way between the two blocks, and for trials where the blocks were held 20 cm above the table (and again 30 cm apart), participants fixated at the point in space 20 cm above the table and mid-way between the two blocks.

**Fig 3:**
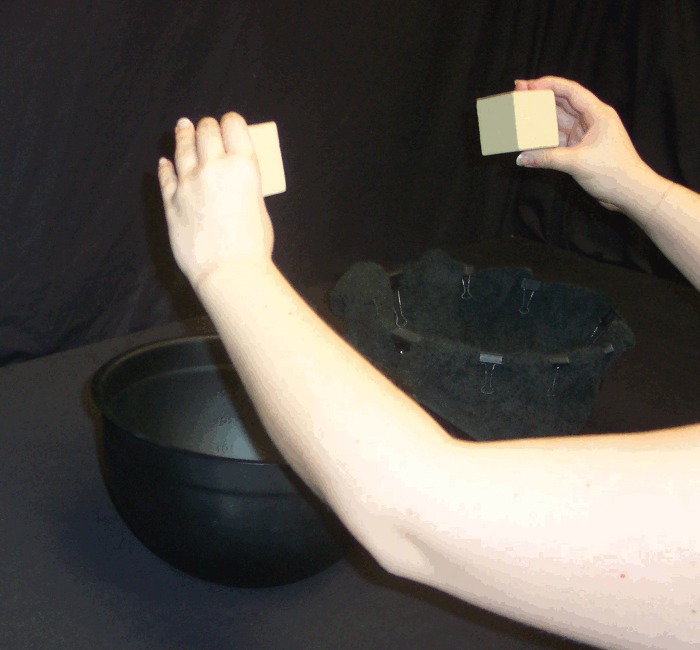
Bowls. Participant holding blocks approximately 20 cm above the bowls.

To control auditory feedback when the wooden blocks were dropped, on some trials participants had two metal bowls (24 cm diameter x 14 cm depth) in front of them, such that the base of each bowl was 30 cm apart and just within arm's reach equidistant in front of the participant. In other words, the bowls were centered at or directly under the same location that the blocks began on each trial, and participants dropped one of the two blocks into one of the two bowls on a given trial while the other block remained stationary. The inner surface of one bowl was padded with a towel to dampen sound when a block was dropped into the bowl (Fig 3). Additionally, for some trials, participants used a pair of black kitchen tongs (30 cm long), rather than their hands, to hold the blocks 30 cm apart and 20 cm above the table at the beginning of a trial. Loosening their squeeze-grip on one tong allowed the participant to drop one of the two held blocks into one of the two bowls on a given trial while the other block remained stationary.

To ensure proper setup for each trial, a small piece of Velcro was affixed to the table where each bowl went and the bowls had a matching small piece of Velcro affixed to the bottom. The experimenter had full view of the table when the flash occurred for each trial (and could hear the dropped objects) and thus could verify that everything started in the correct position.

A Sony IC-Recorder audio recording device recorded participant responses throughout the session, after which the recorded file was transcribed by an experimenter and deleted to retain confidentiality.

### Procedure

Each participant signed a consent form and then the experimenter explained and demonstrated the procedure while the room lights were still on. After this, the lights were turned off and a 10 minute period of dark adaptation followed [25], after which trials began.

Each trial began with the participant placing or holding the blocks in the proper location (and, for relevant conditions, placing the bowls and using the tongs to hold the blocks), fixating their gaze and informing the experimenter that they are ready. Then the experimenter discharged the flash and participants waited for an afterimage of the blocks to fully form, at which point they took action (described below) on one of the blocks while keeping the remaining block stationary. After taking action on one of the blocks (the 'action block'), the participant continued fixating between the starting location of the two blocks and observed (in peripheral vision) the afterimage of the blocks.

**Fig 4:**
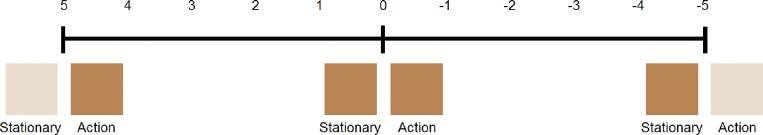
Relative vividness measure. Participants rate the vividness of the action block's afterimage relative to the vividness of the stationary block's afterimage, on an 11-point scale ranging from -5 (much less vivid) to +5 (much more vivid). Adapted from Hogendoorn et al. [10].

Once the entire afterimage had disappeared, the participant verbally reported two measures. First, they gave a binary response whether or not the afterimage of the action block disappeared, faded or crumbled immediately after taking the action. Second, they rated the relative vividness of the afterimage of the action block compared to the afterimage of the stationary block, immediately following the action. This measure (adapted from Hogendoorn et al. [10]) was on an 11-point scale ranging from -5 (afterimage of action block was much less vivid than the afterimage of the stationary block) to +5 (afterimage of the action block was much more vivid), with 0 meaning the afterimages were equally vivid after taking the action (Fig 4). Using both measures minimizes the known issue with asking participants to report only about disappearance or only about visibility of an afterimage [26].

Following this, the participants were encouraged to verbally free-report anything about their experience of the afterimages during that trial. Two minutes of dark adaptation followed before beginning the next trial.

### Design

Participants completed a block of 3 trials for each of 7 conditions. The order of conditions was counter-balanced across subjects in a Latin Square Williams Design (requiring at least 14 participants to balance for first-order carryover effects). Throughout the experiment, each trial alternated which block (left or right) was the action block and which was the stationary block.

*Pull Back*: the participant held a block in each hand 20 cm above the table. After the light flashed and the afterimage formed, the participant pulled one hand (with block in hand) back toward their body and off to the same side, while keeping it at the same horizontal level above the table.

*Drop Loud*: the participant again held the blocks in each hand 20 cm above the table, but with the bowls placed on the table directly underneath (i.e. such that the block was held above the middle of the bowl and could be dropped directly into it). The participant dropped the action block into the bowl that was not lined with a towel (the 'hard bowl'), while keeping the other block stationary above the bowl that was lined with a towel (the 'soft bowl'). The participant was instructed to hold the blocks such that one side of the block was facing them unobstructed by digits (i.e. the blocks are held with thumb underneath and fingers on top so that digits do not obstruct vision of the block).

*Drop Quiet*: this was identical to *Drop Loud*, except that the position of the bowls was reversed such that the action block was dropped into the soft bowl that was lined with a towel, dampening auditory feedback of the dropped block.

*Tong Drop Loud*: this was identical to *Drop Loud*, except that the blocks were held not in the participant's hands but rather in the tongs which were held by the participant directly above each bowl. Participants were instructed to hold the tongs to make contact on the top and bottom of each block so that vision of the side of the block facing the participant was unobstructed by the tongs. Since the tongs extend the functional length of the participant's reach, the participant was instructed to bend their arms out to the side slightly so that the tongs still held each block directly above its respective bowl. In other words, the blocks began the trial at the same physical locations as in the first three conditions, but held by tongs. In this condition, once the afterimage formed, the participant opened the tongs on one side, releasing the action block into the hard bowl.

*Tong Drop Quiet*: this was identical to *Tong Drop Loud*, except that the position of the bowls was reversed such that the action block was dropped into the soft bowl.

**Fig 5:**
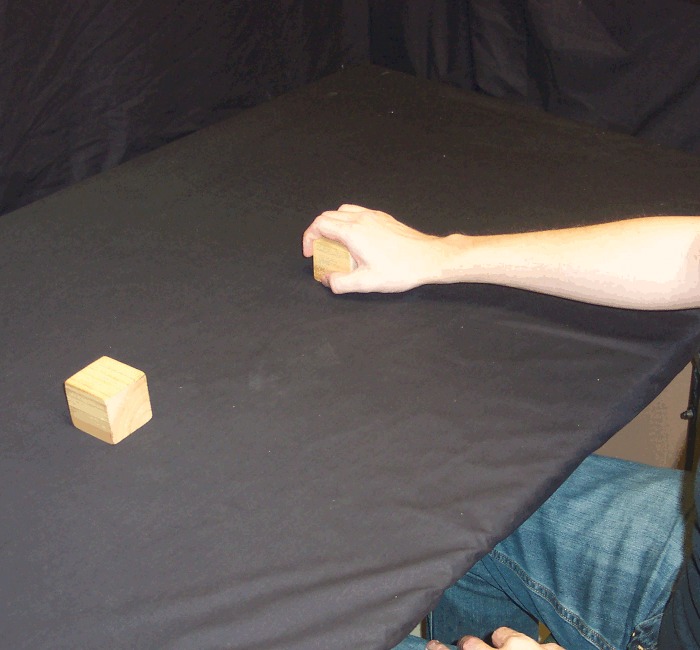
*Reach and Retrieve* condition. Participant reaches out and grabs the action block.

**Fig 6:**
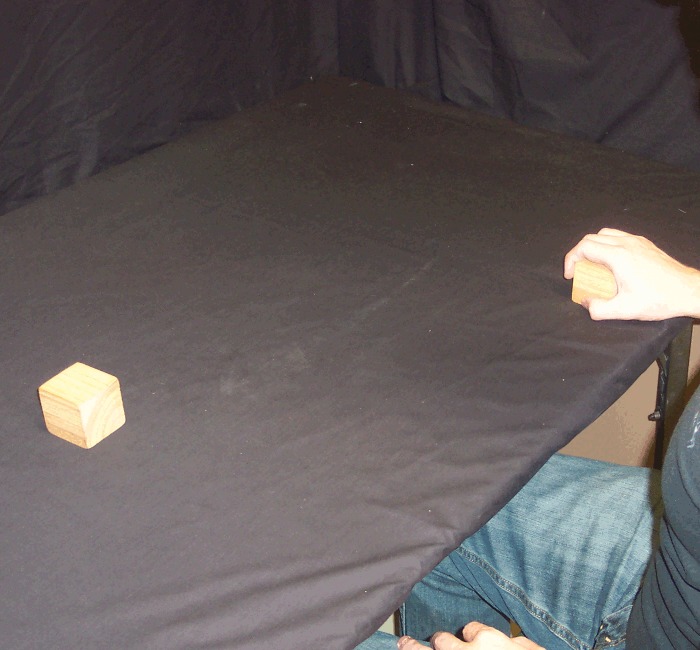
*Reach and Retrieve* condition. Participant then slides the action block back and to the side along the table.

*Reach and Retrieve*: rather than holding the blocks in the air, before these trials the participant placed the blocks on the table 30 cm apart, equidistant from their body and just within reach (i.e. on the table just below where the blocks were held in the air in the first five conditions). In this condition, the participant's hands were not on the table, but held by their side or on their lap, such that the visual scene lit up by the flash (and the subsequent afterimage) contained only the blocks and no hands, bowls or tongs (Fig 1). Once the afterimage formed, the participant reached out with one hand (the hand on the same side as the action block) and grabbed the action block from above, pulling it back along the table surface toward their body and off to the same side (Figs 5 and 6). In other words, the action block's path matched that of *Pull Back*, but on the table rather than above it.

**Fig 7:**
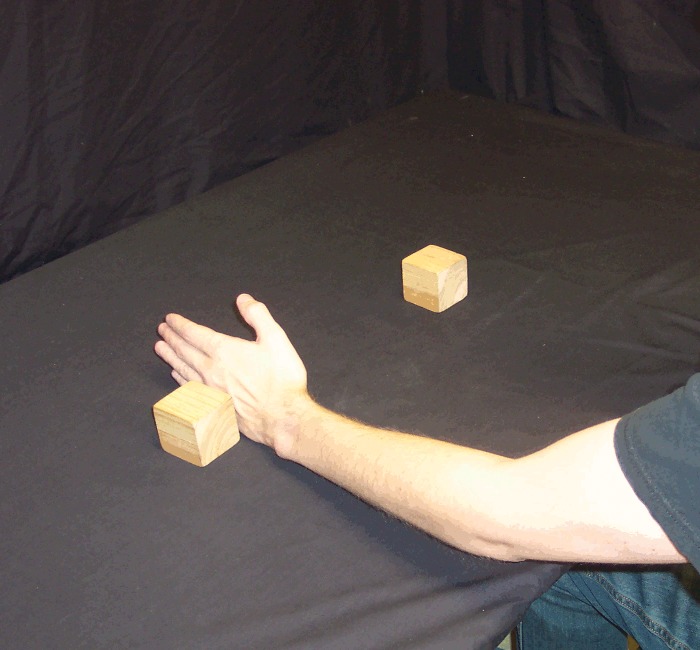
*Reach and Brush Back* condition. Participant reaches out and brushes the block along the table, back and to the side, using the back of the action hand.

**Fig 8:**
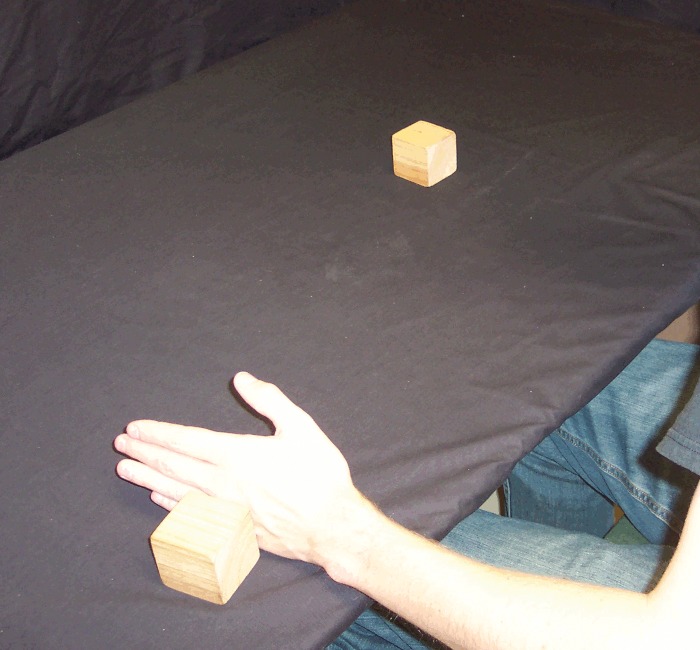
*Reach and Brush Back* condition. Participant reaches out and brushes the block along the table, back and to the side, using the back of the action hand.

*Reach and Brush Back*: this was identical to *Reach and Retrieve*, except instead of reaching out and grabbing the action block from above, the participant reached out with the hand on the same side as the action block (flat, with fingers extended, as for a hand-shake) and used the back of that hand to brush the action block back along the table surface toward their body and off to the same side (Figs 7 and 8). Thus, the action block's path matched that of *Reach and Retrieve*, but rather than move within the hand's grip, it was brushed there with the back of the hand.

*Practice Trials*: before beginning the 7 conditions (3 trials each), the participant also completed 2 practice trials that did not involve any blocks, bowls or tongs, in order to get used to the experience of afterimages and the reporting procedure. In the practice trials, the participant held their hands in front of them 30 cm apart, 20 cm above the table, with palms facing outward (as if to shove something). Once the afterimage of the hands formed, the participant waved one hand up and down while the other hand remained stationary. Then, when the afterimage had faded completely, they were asked to report the same 2 dependent measures taken for the blocks after each trial: (1) immediately after waving the action hand, did the afterimage of the action hand seem to disappear, fade or crumble, and (2) immediately after waving it around, rate the vividness of the afterimage of the action hand relative to the afterimage of the stationary hand on an 11-point scale.

### Predictions

Dependent measures were (1) proportion of trials in which the afterimage of the action block is reported to fade, disappear or crumble after the action is taken (a 'yes' response on a binary 'yes'/'no' question), and (2) vividness of the afterimage of the action block relative to the afterimage of the stationary block, on a -5 to +5 scale.

We predicted that the addition of clear auditory feedback of the block's movement in *Drop Loud* would lead to more fading and less vividness of the dropped block's afterimage, compared to *Drop Quiet*. We predicted that adding auditory information to *Tong Drop Loud* would lead to more fading and less vividness than in *Tong Drop Quiet*. Finally, we predicted no meaningful difference in fading or vividness between *Reach and Retrieve* and *Reach and Brush Back*. As a test of equivalence in vividness reports, we arbitrarily define a meaningful difference as an effect size of at least 1.0 on the -5 to +5 scale; however, we report a confidence interval for the effect size so that the reader may draw their own conclusions as to equivalence.

## Experiment 1—Results

**Fig 9:**
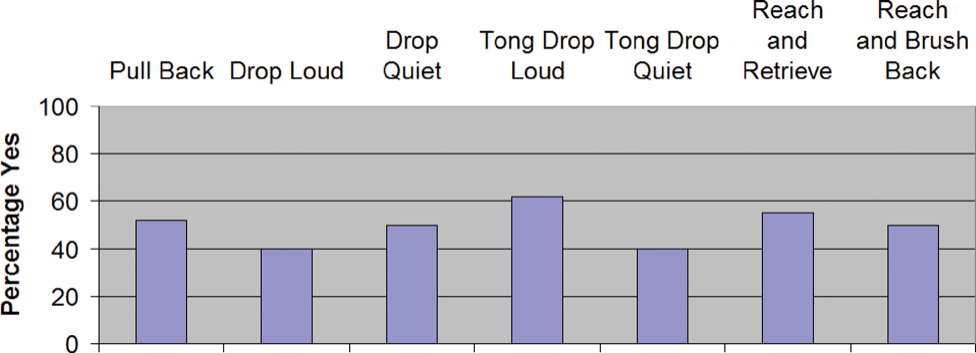
Binary yes/no ratings for Experiment 1 ("Did the afterimage of the action block disappear, fade or crumble immediately after taking the action?").

**Fig 10:**
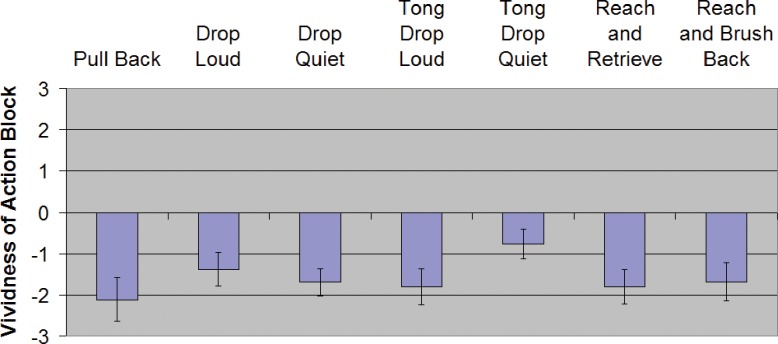
Relative vividness ratings for Experiment 1. **Error bars are SEM.** Y-axis is the reported vividness of the action block relative to the vividness of the stationary block after the condition's action was performed. Negative values mean the action block was less vivid or faded more than the stationary (control) block.

For descriptive statistics in each condition see Table 1 and Figs 9 and 10. For inferential statistics and 95% confidence intervals of effect sizes see Table 2.

**Table 1:** Descriptive statistics for binary measure and vividness measure.

Condition	% yes (95% CI)	Vividness M (SD)
*Pull Back*	52% (38–67%)	-2.12 (1.98)
*Drop Loud*	40% (27–56%)	-1.38 (1.54)
*Drop Quiet*	50% (36–64%)	-1.69 (1.22)
*Tong Drop Loud*	62% (47–75%)	-1.81 (1.64)
*Tong Drop Quiet*	40% (27–56%)	-0.76 (1.36)
*Reach and Retrieve*	55% (40–69%)	-1.81 (1.56)
*Reach and Brush Back*	50% (36–64%)	-1.64 (1.70)

**Table 2:** Inferential statistics.

Comparison	Measure	Inferential Results
*Drop Loud* vs. *Drop Quiet*	% yes	Fisher's exact, P = 0.5111, ns
*Drop Loud* vs. *Drop Quiet*	vividness	Paired t_13_ = 0.6135, P = 0.5501, ns, 95% CI of difference in means: +0.78 to -1.40
*Tong Drop Loud* vs. *Quiet*	% yes	Fisher's exact, P = 0.0802, ns
*Tong Drop Loud* vs. *Quiet*	vividness	Paired t_13_ = 2.7975, P = 0.0151 (but not significant after correction for multiple comparisons), 95% CI of difference in means: -0.24 to -1.86
*Retrieve* vs. *Brush Back*	% yes	Fisher's exact, P = 0.8272, ns
*Retrieve* vs. *Brush Back*	vividness	Paired t_13_ = 0.4140, P = 0.6856, ns, 95% CI of difference in means: -1.04 to +0.70
*Retrieve*	vividness	1-sample t_13_ = 3.6116, P = 0.0032 (still significant after Bonferroni correction)
*Brush Back*	vividness	1-sample t_13_ = 4.3348, P = 0.0008 (still significant after Bonferroni correction)

### Dropping a held object, with and without auditory feedback

Contrary to our predictions, we failed to detect any difference between *Drop Loud* and *Drop Quiet* in either measure used (Fisher's exact, P = 0.5111 for the binary measure and t_13_ = 0.6135, P = 0.5501 for vividness). Adding auditory feedback did not clearly increase the fading or decrease the vividness (although the 95% CI includes values larger than 1.0 on the -5 to +5 scale, so we cannot rule out a difference at least that large).

### Dropping an object held with a tool, with and without auditory feedback

Our participants reported fading in 40% of *Tong Drop Quiet* (95% CI: 27–56%). However, with the vividness measure, *Tong Drop Quiet* averaged a mere -0.76. With clear auditory feedback in *Tong Drop Loud*, the percentage of trials in which participants reported fading was 62% and the average vividness measure was -1.81. In the binary response measure, we failed to detect any significant difference in reported fading between *Tong Drop Loud* and *Tong Drop Quiet* (Fisher's exact, P = 0.0802). In the vividness measure, *Tong Drop Loud* appeared less vivid than *Tong Drop Quiet*, as predicted (t_13_ = 2.7975, P = 0.0151); however, the difference was not significant after correction for multiple comparisons (i.e., P was not < 0.008).

### Moving a held object or a non-held object

In both *Reach and Retrieve* and *Reach and Brush Back*, the participant receives tactile and proprioceptive feedback of the block's movement, yet only in the *Reach and Retrieve* condition is the participant holding the block. As predicted, we failed to detect any difference between *Reach and Retrieve* and *Reach and Brush Back* on either measure (Fisher's exact, P = 0.8272 for the binary measure and t_13_ = 0.4140, P = 0.6856 for vividness), despite both conditions showing significant fading (t_13_ = 3.6116, P = 0.0032, and t_13_ = 4.3348, P = 0.0008). The 95% CI of the difference suggests that any difference between the conditions is likely not more than 1.0 on the -5 to +5 scale.

### Addressing possible side bias

Due to constraints of the testing equipment, the flash was located a little to the left of the participants, rather than directly behind them. To test if this may have created a side bias, for each condition we compared all trials where the block on the left side was the action block to all trials where the block on the right side was the action block. There was no significant difference (Ps > 0.05) in all conditions except *Reach and Retrieve* (P = 0.041), which is not significant when correcting for multiple comparisons.

### Comparison of dependent measures

On trials in which participants reported "no" on the binary measure, the mean vividness rating was -0.36 (CI of effect size: -0.09 to -0.64). The movement-induced fading may not be captured well by a simple binary classification; the more nuanced vividness measure shows that participants perceived an effect of the moved object even when they did not classify the effect as a "yes" response to the binary measure.

## Experiment 1—Discussion

We predicted that adding clear auditory feedback when dropping an object would lead to a stronger afterimage fading effect due to increased conflict between visual information about object location (the illusory afterimage) and non-visual information about object location (the sound). Oddly, when dropping directly from the hands, we did not find a significant difference in either measure when auditory feedback of the drop was added (*Drop Loud* versus *Drop Quiet*), though we also could not rule out a difference of at least 1.0 on the -5 to +5 scale. One plausible explanation is that *Drop Quiet* (and similarly, *Tong Drop Quiet*) did not sufficiently mask auditory feedback (see Experiment 2, below). Alternately, or additionally, direct tactile and proprioceptive feedback from releasing the block may have been sufficient to induce fading (decrease vividness) in both *Drop Loud* and *Drop Quiet*, leading to a ceiling effect where additional auditory feedback did not increase the effect. Finally, this result is also consistent with Carlson et al.'s [13] interpretation that the object was indeed integrated into the body schema when held, and auditory feedback subsequent to dropping the object would not affect this (but see Experiments 2 and 3).

Based on Carlson et al. [13], we predicted that dropping from a gripper tool without auditory feedback would fail to produce fading; their participants reported fading in a mere 4% of trials dropping a piece of wool quietly from a table-supported mechanical gripper (significantly less than the 69–86% in their other conditions). Unlike Carlson et al. [13], we found fading in 40% of trials for *Tong Drop Quiet*. Note that our experiments were performed prior to the publication of Bruggeman et al. [14], whose results did show fading in 80% of trials when dropping from a freely-held tool as opposed to roughly 30% with a table-supported tool (both situations with unknown auditory conditions, but dropping a ball rather than a piece of wool).

While the fact that our participants held the tool freely may present an explanation for why our *Tong Drop Quiet* had more yes responses on the binary response than Carlson et al's [13] participants dropping from a table-supported tool (and thus might even be interpreted as support for a second-order extension of the body schema), it would not explain any differences between *Tong Drop Quiet* and *Tong Drop Loud*—differences which our account does in fact predict. Increasing the sensory information that competes with the illusory visual input of the afterimage should increase the fading effect (either from auditory input alone, or from auditory input being added to the proprioceptive input when dropping from a freely-held tool). In this case, our prediction was not clearly supported (no significant difference detected on the binary measure and the difference in vividness was no longer significant after conservative correction for multiple comparisons). Thus, despite the apparent trend in the data, the tool-mediated dropping results are inconclusive and not discussed further.

Our results were, however, clearly in contrast to the body schema extension account in the comparison between *Reach and Retrieve* and *Reach and Brush Back*. We found no significant difference between these two conditions (and the confidence interval for the effect size suggests any difference that exists is likely not greater than 1.0 on the -5 to +5 scale), despite the fact that in *Reach and Brush Back* the participants were not holding the object at all. Thus, the only way for the body schema incorporation account to accommodate these results is if merely touching objects (as opposed to holding them) is sufficient to quickly incorporate them into the body schema. A sensory updating account seems more parsimonious: despite illusory visual input that the block is where it started, adding tactile and proprioceptive input of a change in the block's spatial location is sufficient to 'overrule' the visual information and cause that afterimage to fade.

Next, we performed a follow-up experiment in which we added a control condition with no block movement; this allowed us to rule out the possibility that drawing spatial attention to one side is sufficient to cause afterimage fading. In order to disentangle the possible explanations for an unexpected result in Experiment 1 (where *Drop Loud* showed no less vividness than *Drop Quiet*), we also repeat *Drop Loud* but add a *Drop Silent* condition where all auditory information is removed.

## Experiment 2—Method

### Participants

Twelve healthy adult undergraduate and graduate student volunteers (7 female, 5 male) participated in this experiment. Age ranged from 22 to 32 years (mean = 26.5). Eleven were right handed and all had normal or corrected-to-normal vision. Participants were asked to remove any watches and jewelry from their hands and wrists prior to starting the experiment. All participants were naïve as to the hypotheses of the experiment. All research was approved by the Institutional Review Board of the University of Georgia; each participant signed a consent form and was debriefed after the study.

### Materials and apparatus

The materials were the same as in Experiment 1 except that the tongs were not used and in one condition participants wore noise-canceling headphones (Bose QuietComfort 15) playing a continuous track of white noise to remove all auditory feedback.

### Procedure

The procedure was the same as in Experiment 1 except that relative vividness was the only dependent measure taken. The binary response about the disappearance of the action block's afterimage was removed to ensure it was not biasing participants into expecting disappearance and because it appears from Experiment 1 that participants report "no" even when movement does make the afterimage on the moved side less vivid.

### Design

Participants completed a block of 3 trials for each of 4 conditions. The order of conditions was counter-balanced across subjects in a Latin Square Williams Design (requiring at least 4 participants to balance for first-order carryover effects). Throughout the experiment, each trial alternated which block (left or right) was the action block and which was the stationary block.

*Pull Back*: identical to *Pull Back* in Experiment 1.

*Hold in Place*: identical to *Pull Back*, except that participants held both blocks in place once the afterimage had formed. Neither block was moved, and participants were instructed to simply rate the vividness of the target block's afterimage relative to the other one. This served as a control for *Pull Back*, and also allowed us to test if spatial attention being drawn to one side was sufficient to induce the disappearance of that afterimage without any actual movement taking place.

*Drop Loud*: identical to *Drop Loud* in Experiment 1.

*Drop Silent*: identical to *Drop Quiet* in Experiment 1, except that participants wore noise-canceling headphones playing white noise. Thus, auditory information of the block being dropped was completely removed, rather than just dampened.

*Practice Trials*: identical to Experiment 1, except that participants only rated the relative vividness of the action hand.

### Predictions

We predicted that *Pull Back* would again lead to relative vividness significantly lower than 0, but that *Hold in Place* would not, and that *Pull Back* would show significantly lower vividness than *Hold in Place*.

We also predicted that *Drop Loud* would show relative vividness significantly lower than *Drop Silent*. Under the body schema extension account, it is not clear why adding sounds subsequent to releasing a held object should make the dropped object more integrated into the body schema; on other hand, our account clearly predicts that dropping an object should cause the afterimage to disappear if there is sufficient non-visual sensory input to compete with the visual input of the afterimage.

## Experiment 2—Results

**Fig 11:**
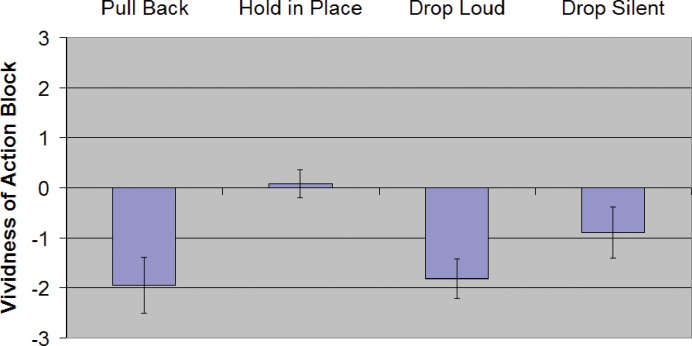
Relative vividness ratings for Experiment 2. **Error bars are SEM.** Y-axis is the reported vividness of the action block relative to the vividness of the stationary block after the condition's action was performed. Negative values mean the action block was less vivid or faded more than the stationary (control) block.

For descriptive statistics in each condition see Table 3 and Fig 11. For inferential statistics and 95% confidence intervals of effect sizes see Table 4.

**Table 3:** Descriptive statistics.

Condition	Vividness M (SD)
*Pull Back*	-1.94 (1.96)
*Hold in Place*	+0.08 (0.94)
*Drop Loud*	-1.81 (1.37)
*Drop Silent*	-0.89 (1.77)

**Table 4:** Inferential statistics.

Comparison	Inferential Results
*Pull Back* vs. *Hold in Place*	Paired t_11_ = 2.880, P = 0.0150 (still significant after Bonferroni correction), 95% CI of difference in means: -3.58 to -0.48
*Drop Loud* vs. *Drop Silent*	Paired t_11_ = 2.407, P = 0.0348 (not significant after Bonferroni correction, but still significant after Hochberg correction), 95% CI of difference in means: -1.76 to -0.08

### Moving or not moving a block

The *Pull Back* condition showed significantly lower vividness ratings than the *Hold in Place* control condition (t_11_ = 2.880, P = 0.0150).

### Removing all auditory feedback

With auditory feedback completely removed, we found lower vividness ratings in *Drop Loud* than in *Drop Silent* (t_11_ = 2.407, P = 0.0348).However, with the most conservative correction for multiple comparisons, the results may not be significant, so these data should be interpreted with caution.

## Experiment 2—Discussion

The findings in Experiment 2 strengthen our earlier findings by adding a control condition (*Hold in Place*) and by testing a possible explanation for unexpected findings in Experiment 1 (where *Drop Loud* showed no lower vividness than *Drop Quiet*). Data from *Hold in Place* provide further evidence that participants are not simply giving lower ratings of vividness to the action block in every condition. Furthermore, this condition suggests it is unlikely spatial attention is the primary driver of the afterimage fading effect.

Results from *Drop Loud* and *Drop Silent* suggest that auditory feedback does make a significant difference, and suggest that the small amount of auditory feedback in *Drop Quiet* may have contributed to the unexpected results in Experiment 1. It is not clear how body schema extension would account for these results, where the afterimage is affected by auditory feedback occurring well after physical contact has ceased.

While the data above provide tentative evidence for the role of auditory information in the afterimage fading effect, these data are unable to tease apart the relative contribution or necessity of proprioceptive, tactile, or reafferent signals. Hogendoorn et al. [10] showed that proprioception, rather than reafferent motor signals, drive the afterimage fading effect for a body part which is moved in the dark, but in the case of held objects we must still find a way to test the effects of proprioception independent of touch and touch independent of proprioception. That was the aim of our third experiment. In one set of conditions, we manipulated the mass of an object dropped in the dark while holding other sensory information constant. If the afterimage fading effect scales with the strength of proprioceptive feedback, it would provide evidence that proprioception plays a role above and beyond the effects of touch. In another set of conditions, we manipulated tactile information while holding other sensory information constant. If the afterimage fading effect still occurs, it will provide evidence that tactile information alone is sufficient to capture vision, and will provide further evidence that the fading of a moved object's afterimage is independent of the object being held (and hence, unlikely to be explained as an effect of held objects being assimilated into the body schema).

## Experiment 3—Method

### Participants

The participants were eleven healthy adult undergraduate and graduate student volunteers (5 female, 6 male). Age ranged from 19 to 34 years (mean = 26.5). Ten were right handed and had normal or corrected-to-normal vision and were naive as to the hypotheses of the experiment. Participants removed any watches and jewelry from their hands and wrists for the duration of the experiment. All research was approved by the Institutional Review Board of the University of Georgia; each participant signed a consent form and was debriefed after the study.

### Materials and apparatus

For each trial, a pair of cubic blocks (5 x 5 x 5 cm) was either held by the participant 20 cm above the table (Conditions 1–3) or sat in the participant's upturned palms as their arms rested on the table (Conditions 4–5). In both cases, the blocks began each trial approximately 30 cm apart, equidistant in front of the participant. In Condition 1, the blocks were made of styrofoam covered in two layers of dark tan masking tape and weighed 10 grams. In Conditions 2, 4 and 5, the blocks were made of wood covered in two layers of dark tan masking tape and weighed 60 grams. In Condition 3, the blocks were made of dense clay covered in two layers of dark tan masking tape and weighted 260 grams. Thus, all blocks looked the same but differed dramatically in mass.

In Conditions 1–3 participants placed the bowls from Experiment 2 in front of them as before and participants dropped one of the two blocks into one of the two bowls on a given trial while the other block remained stationary. Both bowls were black and non-reflective, and the inner surface of each bowl was padded with a suspended black towel to catch the falling block and dampen sound when a block was dropped into the bowl.

Additionally, for all conditions participants wore noise-canceling headphones (Bose QuietComfort 15) playing a continuous track of white noise to remove all auditory feedback.

### Procedure

**Fig 12:**
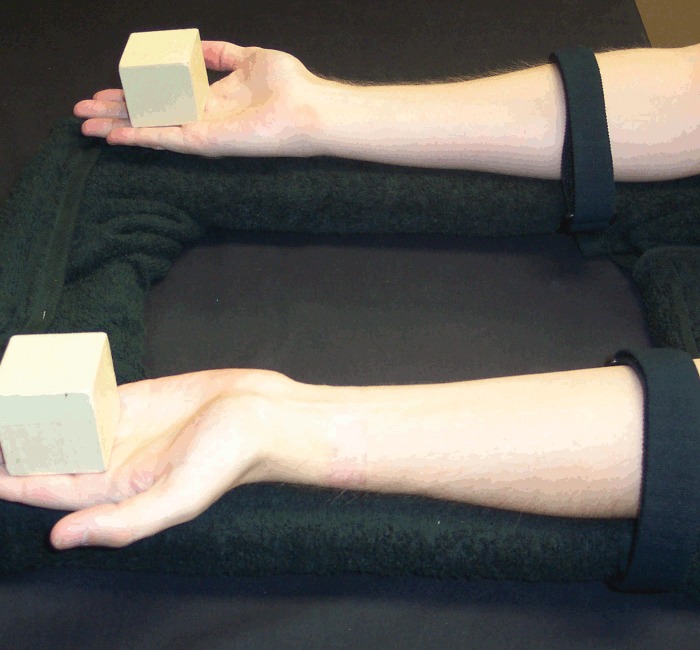
Blocks resting on participant's hands. In Condition 4, an experimenter slid the action block down the participant's arm to the Velcro strap. In Condition 5, neither block was moved.

**Fig 13:**
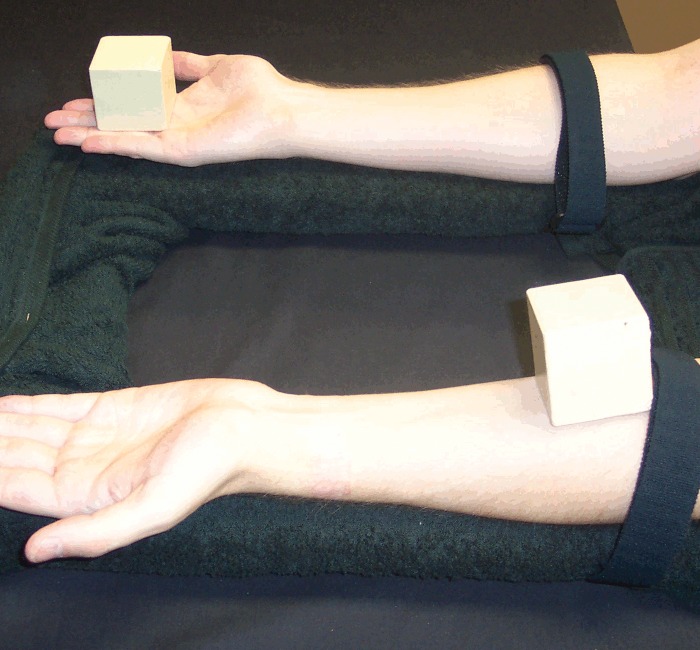
Action block's location at the end of Condition 4 trials.

For Conditions 1–3, the procedure was the same as in Experiment 2: once the afterimage fully formed, the participant dropped one of the blocks and then reported the relative vividness measure. For Conditions 4–5, a second experimenter stood to the side of the table, and in Condition 4 (Figs 12 and 13), once the afterimage had formed, that experimenter slid one block down the participant's palm, wrist and forearm, stopping at a strap just before the elbow (in Condition 5, neither block moved).

### Design

The order of the five conditions was counter-balanced across subjects in a Latin Square Williams Design (requiring at least 10 participants to balance for first-order carryover effects). Participants completed 3 trials of each condition, and throughout the experiment, each trial alternated which block (left or right) was the target block and which was the stationary block.

Condition 1, *Drop Styrofoam*: identical to *Drop Silent* in Experiment 2, but using the styrofoam blocks.

Condition 2, *Drop Wood*: identical to *Drop Styrofoam*, but using the wooden blocks.

Condition 3, *Drop Clay*: identical to *Drop Styrofoam*, but using the clay blocks.

Condition 4, *Sliding*: the participant rested their arms on the table with their hands palms-up and completely open, and with a wooden block resting on each palm. Participants' arms were held in place by a Velcro strap attached to the table and looping around their forearm close to the elbow. Following the formation of the afterimage, the participant took no action, but the second experimenter slid the target block down the participant's entire palm, wrist and forearm until it reached the strap near the elbow.

Condition 5, *Stationary*: identical to *Sliding*, but in this case the experimenter did not move the target block. Instead, the participant simply waited one second after the afterimages had formed, and then rated the relative vividness of the target block relative to the stationary block.

*Practice Trials*: identical to Experiment 2.

### Predictions

In the *Drop* conditions (*Styrofoam*, *Wood*, *Clay*), the visual input was equivalent, and there was no auditory feedback. Greater mass (and thus greater change in torque upon release) should provide greater proprioceptive feedback of the change in the block's spatial position after dropping it. Thus, proprioceptive input about the block's position is in greatest conflict with the (illusory) visual input of the afterimage in the *Drop Clay* condition, intermediate in the *Drop Wood* condition, and in least conflict in the *Drop Styrofoam* condition. Thus, we predicted significantly lower vividness ratings in the *Drop Clay* condition than the *Drop Wood* condition, and significantly lower ratings in the *Drop Wood* condition than the *Drop Styrofoam* condition.

In the *Sliding* and *Stationary* conditions, there is no difference in visual, auditory, proprioceptive or motor input, only in tactile input. In *Sliding*, there is tactile feedback of the block's change in spatial location, whereas in *Stationary*, no sensory input conflicts with vision. Thus, we predicted significantly lower vividness ratings in *Sliding* than in *Stationary*.

## Experiment 3—Results

**Fig 14:**
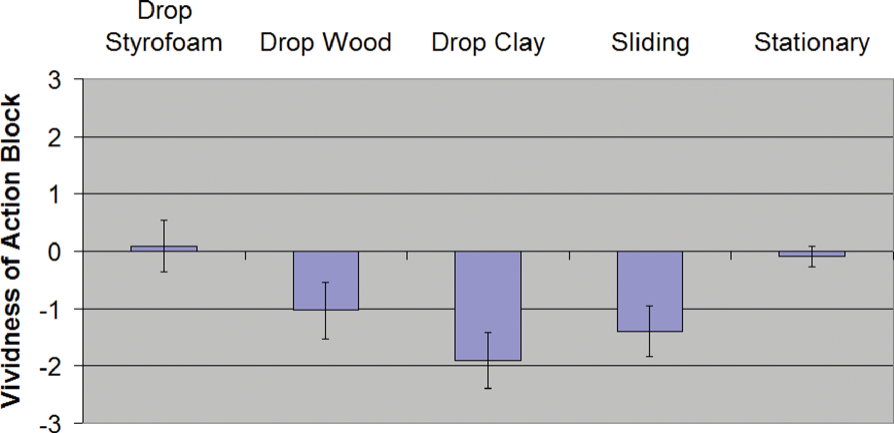
Relative vividness ratings for Experiment 3. **Error bars are SEM.** Y-axis is the reported vividness of the action block relative to the vividness of the stationary block after the condition's action was performed. Negative values mean the action block was less vivid or faded more than the stationary (control) block.

For descriptive statistics in each condition see Table 5 and Fig 14. For inferential statistics and 95% confidence intervals of effect sizes see Table 6.

**Table 5:** Descriptive statistics.

Condition	Vividness M (SD)
*Drop Styrofoam*	+0.09 (1.47)
*Drop Wood*	-1.03 (1.64)
*Drop Clay*	-1.91 (1.61)
*Sliding*	-1.39 (1.46)
*Stationary*	-0.09 (0.58)

**Table 6:** Inferential statistics.

Comparison	Interferential Results
*Styrofoam / Wood / Clay*	W/IN SUBJ ANOVA, F_2,20_ = 14.173, P < 0.001, (still significant after Bonferroni correction)
*Styrofoam* vs. *Wood*	post-hoc t = 2.976, P = 0.014, 95% CI of effect: +0.28 to +1.96
*Styrofoam* vs. *Clay*	post-hoc t = 4.205, P = 0.002, 95% CI of effect: +0.94 to +3.06
*Wood* vs. *Clay*	post-hoc t = 3.677, P = 0.004, 95% CI of effect: +0.35 to +1.41
*Sliding* vs. *Stationary*	Paired t_10_ = 2.922, P = 0.0153 (still significant after Bonferroni correction), 95% CI of effect size: -2.30 to -0.31

### Effect scales with magnitude of proprioceptive change

We found a significant difference in vividness ratings between the conditions dropping objects of different mass (F_2,20_ = 14.173, P < 0.001), and all pair-wise comparisons were significant. The heavier the object that is dropped, the greater the change in torque; this corresponds to more proprioceptive feedback [27], which appears to strengthen the afterimage fading effect.

### Passive tactile feedback is sufficient to induce fading

As predicted, vividness ratings in *Sliding*, where the object was moved by the experimenter, were significantly lower than in *Stationary* (t_10_ = 2.922, P = 0.0153).

## Experiment 3—Discussion

The results in the *Drop* conditions clearly demonstrate that proprioceptive feedback matters above and beyond tactile feedback (which was constant) or auditory feedback (which was completely removed).

Significant fading in *Sliding* shows that the afterimage fading effect can be induced without any auditory, proprioceptive, or reafferent motor feedback. Like Hogendoorn et al. [10], this shows that active bodily movement and reafferent motor signals are not necessary for afterimage fading. Indeed, no bodily movement at all is necessary. Furthermore, the results in *Sliding* are in line with Experiment 1 (*Reach and Brush Back* condition) in showing that the effect can happen without the participant holding the object at all, and so a body schema integration account seems unlikely.

## General Discussion

The three experiments presented here provide clear evidence that the visual percept of a positive afterimage is clearly affected by information from the non-visual senses, and that when the senses provide conflicting information about the spatial position of an object in the dark, vision may be captured by sufficient input from the auditory, tactile and proprioceptive modalities. This sensory capture for afterimages of objects moved in the dark does not rely on the object being held or moved by the observer, and need not involve incorporation of the object into the body schema. While previous explanations of afterimage disappearance related to multi-sensory processing of the body's parts to track limbs in space [8, 10], this work demonstrates that disappearance of an afterimage of an object that is moved is a general phenomenon of which the processing of afterimages of body parts may be a special case.

Furthermore, these experiments clearly show that multiple non-visual senses all contribute to the effect. We found tentative evidence that adding auditory feedback of object movement may be sufficient to induce fading. We also demonstrated that tactile feedback alone is sufficient to induce the fading effect, and that proprioception plays an important role above-and-beyond tactile or auditory information.

Future psychophysical work could further tease apart the relative contributions of the individual sensory systems (i.e. the precise amount of stimulation required in a given sense to over-ride an afterimage of a given strength). This work generates other questions for future study. For example, Dieter et al. [28] demonstrate visual percepts apparently evoked by self-generated movement, presumably due to predictive associations from the senses normally matching. If participants in the current testing paradigm are trained to associate an arbitrary sound with actively moving a block in the dark (subsequent to afterimaging), would the afterimage still disappear on probe trials when sound is hidden during block movement or probe trials where the sound plays without the block moving? And while the tactile and proprioceptive senses (and possibly also audition) have been shown sufficient to capture vision in this paradigm, what about new visual information over-riding the afterimage? Would new visual input that conflicts with the visual afterimage be sufficient to over-ride the afterimage? Future experiments could mark the observer's hand with luminous paint, induce an afterimage, and then use a prism to change the visual field location of the luminous hand without changing the afterimage location.

The sensory updating account presented here proposes a general mechanism—rather than one specific to bodily processing—of the multi-sensory tracking of object location even in darkness. This fits well with an existing parallel literature on size-constancy in afterimages. It has been known for over a century that the perceived size of an afterimage in a lit room is a function of (1) the portion of the visual field it covers and (2) the distance of the surface or plane on which it is 'projected' [29]. In other words, when visual size and distance cues are available, the afterimage is perceived as 'projected' at a particular distance, and size-constancy mechanisms cause it to be perceived as the appropriate size for an object at that distance that takes up that much of the visual field. This effect is called Emmert's law, and interestingly, it works in the dark as well [30]. Suzuki [31], for example, induced an afterimage to an observer in darkness and then manipulated the distance of a point-light target (thus, close-up targets increase convergence). Observers judged the perceived size of the afterimage (which always subtended the same visual angle) as larger when the point-light was closer, and smaller when it was farther, but only up to a limit of about 200 cm (the apparent limit for oculomotor cue information for distance perception), suggesting that what is perceived visually is affected by other sensory/motor information. Likewise, Carey and Allan [32] had observers look at their hand in darkness, then flashed a light to induce an afterimage of the hand, and then either the observer or the experimenter moved that hand forward or backward in the dark. Regardless of whether the movement was passive or active, the apparent size of the hand increased when moved toward the observer and decreased when moved away, suggesting proprioceptive cues influenced the visual perception of the afterimage (in this case, by influencing the distance at which it was perceived). This effect seems to extend (for the most part) to an object held and subsequently moved toward or away from the observer in darkness [33–34].

Why does the afterimage of a body part or object disappear when moved in some experiments [the present study, 8, 10, 13], but change size without disappearing in these other size-constancy experiments (or at least, disappearance was not measured or mentioned in those studies)? The likely explanation is that the non-visual cues provided in these size-constancy experiments don't contradict the visual information (from the afterimage) about the spatial location of the object; rather, those cues are used to determine at which distance the visual afterimage is 'projected' or perceived. If the movements in the darkness were not just toward or away from the observer, but also up/down or to the side, we predict that the afterimage would still fade.

Our explanation for the afterimage fading effect requires a general object-tracking (not just body-tracking) mechanism that works even in darkness. The object permanence and invisible displacement literature is filled with evidence that we can track objects that are out-of-sight (say, when occluded [35–36]), so it should not be surprising if that system turns out to also work when the out-of-sight conditions are due to darkness [37]. Indeed, Graziano et al. [38] present evidence of a specific neural mechanism for this very ability. They identified bimodal neurons in the ventral premotor cortex representing nearby reachable space (or perhaps tracking prepared motor actions into that space); that is they found neurons that fired for tactile stimuli on the arm or for visual stimuli in the space around or reachable by the arm. Not surprisingly, these neurons fired for an object presented within their visual receptive field; however, a subset of these neurons continued to fire even after the room was plunged into darkness. The spatial location was represented, rather than the visual field location, because the visual receptive field remapped with head movement. Hulme & Zeki [39] show with fMRI that the ventral premotor cortex processes occluded objects regardless of occlusion method. Additionally, the ventral premotor cortex also has bimodal and trimodal neurons with auditory receptive fields specifying various distances and directions for sounds near the body, and these work for stimuli presented in darkness [40]. Together, these studies provide evidence of a multi-sensory neural mechanism for tracking the spatial location of an object (within reachable space, at least) even in total darkness, and this mechanism (or neurons with similar properties outside the premotor cortex) could easily underlie the afterimage fading effect even for objects which are not integrated into the body schema.

To summarize, the work presented herein is significant for a number of reasons. We show that recent claims of held objects being rapidly incorporated into the body schema in this afterimage paradigm may be premature. We present evidence for a more general mechanism behind the afterimage fading effect: sensory updating of the brain's representation of the spatial location of an object that is moved in the dark. These experiments tease apart the contribution of different sensory systems to the afterimage fading effect, establishing a clear role for tactile and proprioceptive information, and tentative evidence for the contribution of audition. This clarifies our basic understanding of positive afterimages and provides further evidence that the positive afterimage percept is not due to primarily peripheral activity but constructed centrally in conjunction with information from non-visual senses. Explicating the actual multi-sensory mechanisms underlying object-tracking (including body-part tracking) can, in the future, provide a solid basis on which to build a more nuanced understanding of how the brain's flexibility in terms of body schema might be exploited to create situations where tools are actually incorporated into the body schema.

## Supporting Information

S1 Data Excel spreadsheet with individual data. (XLSX)
